# Responses to High-Fat Challenges Varying in Fat Type in Subjects with Different Metabolic Risk Phenotypes: A Randomized Trial

**DOI:** 10.1371/journal.pone.0041388

**Published:** 2012-07-23

**Authors:** Susan J. van Dijk, Marco Mensink, Diederik Esser, Edith J. M. Feskens, Michael Müller, Lydia A. Afman

**Affiliations:** 1 Division of Human Nutrition, Wageningen University, Wageningen, The Netherlands; 2 Netherlands Nutrigenomics Centre, TI Food and Nutrition, Wageningen, The Netherlands; Paris Institute of Technology for Life, Food and Environmental Sciences, France

## Abstract

**Background:**

The ability of subjects to respond to nutritional challenges can reflect the flexibility of their biological system. Nutritional challenge tests could be used as an indicator of health status but more knowledge on metabolic and immune responses of different subjects to nutritional challenges is needed. The aim of this study was to compare the responses to high-fat challenges varying in fat type in subjects with different metabolic risk phenotypes.

**Methodology/Principal Findings:**

In a cross-over design 42 men (age 50–70 y) consumed three high-fat shakes containing saturated fat (SFA), monounsaturated fat (MUFA) or n-3 polyunsaturated (PUFA). Men were selected on BMI and health status (lean, obese or obese diabetic) and phenotyped with MRI for adipose tissue distribution. Before and 2 and 4 h after shake consumption blood was drawn for measurement of expression of metabolic and inflammation-related genes in peripheral blood mononuclear cells (PBMCs), plasma triglycerides (TAG), glucose, insulin, cytokines and ex vivo PBMC immune response capacity. The MUFA and n-3 PUFA challenge, compared to the SFA challenge, induced higher changes in expression of inflammation genes *MCP1* and *IL1β* in PBMCs. Obese and obese diabetic subjects had different PBMC gene expression and metabolic responses to high-fat challenges compared to lean subjects. The MUFA challenge induced the most pronounced TAG response, mainly in obese and obese diabetic subjects.

**Conclusion/Significance:**

The PBMC gene expression response and metabolic response to high-fat challenges were affected by fat type and metabolic risk phenotype. Based on our results we suggest using a MUFA challenge to reveal differences in response capacity of subjects.

**Trial Registration:**

ClinicalTrials.gov NCT00977262

## Introduction

In the western world food is generally continuously available and most of the day is spent in the postprandial state. Food intake can elicit a transient metabolic and low inflammatory response, especially when high fat is consumed [1], [2], [3]. The magnitude of this response reflects the ability of the biological system to adequately respond to nutrient intake. The presence of metabolic risk phenotypes such as obesity and type 2 diabetes might affect this ability as shown by elevated postprandial triglyceride concentrations in these subjects, in addition to metabolic abnormalities and chronic inflammation in the fasting state [4], [5], [6], [7], [8]. Not only the presence of overall obesity, but also body fat distribution, i.e. increased intra-abdominal (visceral) adipose tissue, might affect the metabolic condition and the postprandial triglyceride response [9].

Elevated postprandial triglyceride concentrations are considered as risk factors for cardiovascular disease and were higher associated with cardiovascular events compared to fasting triglyceride concentrations [10], [11]. The response to a nutritional challenge test might thus be considered as a better biomarker of health status than fasting measures, since this will reflect a person’s metabolic flexibility and capacity to adapt [12], [13]. A widely applied example of a nutritional challenge test is the oral glucose tolerance test for measuring glucose clearance capacity. Nutritional challenge tests might be useful to detect small changes in health status, which could be of major importance in early detection and prevention of disease, but could also be used to test the effectiveness of (nutritional) interventions [12], [14]. However, at the moment relatively little is known about responses of different types of subjects to nutritional challenges such as a high-fat load which makes it difficult to use the response to challenges as biomarkers for characterization of health status.

Postprandial meal studies showed that the type of fat consumed could affect the metabolic and inflammatory response [2], [15], [16], [17], [18], [19] and gene expression responses in circulating peripheral blood mononuclear cells (PBMCs) [18]. PBMCs are immune cells that have been shown to be metabolically active [20], moreover, gene expression profiles of PBMCs were shown to reflect metabolic disease state and changes in nutrient intake which makes these cells an interesting target to investigate responses to nutritional challenges in different subjects [21], [22], [23].

The objective of this study is to characterize the PBMC, metabolic and immune response to high-fat challenges in subjects with different metabolic risk phenotypes. Responses to different types of fat (SFA, MUFA and n-3 PUFA) were compared in order to reveal which type of fat may be best used in future high-fat challenges to test response capacity of subjects.

## Methods

The protocol for this trial and CONSORT checklist are available as supporting information (Protocol S1 and Checklist S1).

### Study Population and Eligibility Criteria

Male Caucasian volunteers aged 50–70 years participated in the study. The study population consisted of three groups; 1) lean subjects, 2) obese subjects and 3) obese type 2 diabetic subjects. Subjects were excluded if they were vegetarian, regular tobacco smoker, allergic to dairy products or fish oil, current or recent user of fish oil supplements, consumed more than four times fish/wk, had an unstable body weight, used antibiotics or anti-inflammatory medication or had a long-term medical condition that could interfere with the study outcome. Lean and obese subjects were excluded when using cholesterol-lowering medication; and lean subjects were excluded when using blood pressure lowering medication. Obese diabetic subjects were all diagnosed with diabetes mellitus in the past, and did not use insulin and/or thiazolidinediones. During a screening visit an oral glucose tolerance test (OGTT) was performed and fasting urinary glucose concentration was measured in healthy subjects to exclude the presence of (unknown) diabetes mellitus (fasting blood glucose <7 mmol/L, 2 hr after OGTT <7.8 mmol/L). Subjects were informed about the design and purpose of the study and all subjects provided written informed consent. The Medical Ethical Committee of Wageningen University (the Netherlands) approved the study and the study was registered at clinicaltrials.gov as NCT00977262. The study was conducted according to the principles of the declaration of Helsinki and in accordance with the Medical Research Involving Human subject Act.

Sample size was determined based on the sample size in a comparable study with lean male subjects in which differences in PBMC gene expression after consumption of three different high fat shakes were detected [18].

### Study Design

All subjects consumed three high-fat shakes enriched with SFA, MUFA or n-3 PUFA in a crossover design, on three different days with at least one week between each study day. Participants in each subject group were randomly allocated to one of the six possible sequences of shake consumption. The possible sequences were equally distributed over the participants within a subject group. A research assistant generated the random allocation sequence and assigned the subjects to the interventions. Shakes were given a letter code and both subjects and researchers were blinded to the intervention.

The evening before the study day, subjects consumed an identical low-fat meal and were not allowed to eat or drink anything after 8 pm except water. The next morning, subjects came to Wageningen University, the Netherlands, and a fasting blood sample was collected. Blood was drawn into EDTA-containing tubes for plasma isolation and into BD Vacutainer Cell Preparation Tubes containing sodium citrate (Becton Dickinson, Breda, Netherlands) for PBMC isolation. After the first blood sample subjects received a high-fat shake, which they had to consume within 15 min. Blood samples were collected 2 h and 4 hours after consumption of the high-fat shake. During a study day, subjects were physically inactive and did not eat or drink anything except water.

### Shake Composition

All high-fat shakes were isocaloric and differed only in fat composition. The shakes contained low-fat yoghurt, low-fat milk, strawberry flavour, 7.5 g of sugar and 95 g of the test fat. The SFA shake contained 95 g palm oil (Research Diet Services BV, Wijk bij Duurstede, The Netherlands) and the MUFA shake contained 95 g high-oleic acid sunflower oil (Aldoc BV, Schiedam, The Netherlands). The n-3 PUFA shake contained 40 g palm oil and 55 g Marinol D-40 (Lipid Nutrition, Wormerveer, The Netherlands), of which 40% was docosahexanoic acid (DHA). Vitamin E (165 mg Tocoblend L50, Vitablend, Wolvega, The Netherlands) was added to Marinol D-40 by the manufacturer to prevent oxidation. The same amount of vitamin E was added to the SFA and MUFA shakes. The macronutrient composition of the shakes was calculated based on the database of the Dutch Nutrient Databank and shown in table 1.

**Table 1:** Macronutrient composition of the fat shakes.

	SFA shake	MUFA shake	n-3 PUFA shake
Energy (kJ)	4074	4074	4074
Energy (kcal)	987	987	987
Protein (g)	10	10	10
Carbohydrates (g)	22	22	22
Fat (g)	95	95	95
SFA (g)	51	8	32
MUFA (g)	37	79	25
PUFA (g)	6	8	38
EPA (g)	–	–	3
DHA (g)	–	–	23
Vitamin E (mg)	165	165	165

### Primary Study Outcomes

The primary outcomes of the study were postprandial gene expression changes of immune-related genes (*IL1b, IL8,* Monocyte chemotactic protein 1 (*MCP1*), nuclear factor of kappa light polypeptide gene enhancer in B-cells 1 (*NFκB1), TNFα*) and lipid metabolism related genes (ATP-binding cassette sub-family A member 1 (*ABCA1),* pyruvate dehydrogenase kinase isozyme 4 (*PDK4),* sterol regulatory element binding transcription factor 1 (*SREBP1),* LDL receptor (*LDLr),* liver X receptor alpha *(LXRα),* cytochrome P450 family 27 subfamily A polypeptide 1 (*CYP27A1*) in PBMCs. These genes were selected because we previously showed that their expression was affected by high-fat consumption [18].

#### PBMC RNA isolation, cDNA synthesis and q-PCR

PBMCs were isolated from whole blood by using BD Vacutainer Cell Preparation Tubes according to the manufacturer’s instructions. PBMC RNA was isolated by using the Qiagen RNeasy Micro kit (Qiagen, Venlo, Netherlands) and reverse transcribed using a cDNA synthesis kit (Promega, Leiden, Netherlands). Standard Q-PCR was performed using SensiMix real-time PCR reagents (Bioline, London, United Kingdom) and a Bio-Rad CFX384 machine (Bio-Rad Laboratories BV, Veenendaal, the Netherlands). Primer sequences used were chosen based on the sequences available in PRIMERBANK (http://pga.mgh.harvard.edu/primerbank/index.html). Q-PCR data were normalized by measuring cycle threshold ratios between candidate genes and a housekeeping gene, human ribosomal protein LP0, which was shown to be consistent within PBMCs [24].

### Secondary Study Outcomes

Secondary study outcomes were change in plasma cytokine, glucose, insulin, triglyceride, and free fatty acid concentration after the high fat challenges. We also studied PBMC immune response capacity before and after the high fat challenges and we determined whether abdominal fat distribution of the subjects influenced the primary and secondary study outcomes.

#### Plasma cytokines

Plasma samples were analyzed on preformatted arrays (pro-inflammatory panel II, Meso Scale Diagnostics, LLC) on a SECTOR Imager 2400 reader (Meso Scale Diagnostics, LLC) for the measurement of IL1β and TNFα.

#### Plasma glucose, insulin, triglycerides, free fatty acids

Immediately after blood was drawn in EDTA-containing tubes it was centrifuged (750×g, 4°C, 10 min), and plasma was stored at −80°C until analysis. Plasma FFA concentrations were analysed by the ACS-ACOD Method (NEFA HR kit, Wako Chemicals CmbH, Neuss, Germany). Plasma triglyceride and glucose concentrations were measured using the Dimension Clinical Chemistry System (Dade Behring Inc, USA). Glucose was measured by the Synchron LX20 System using hexokinase and glucose-6-phosphate dehydrogenase (Glucose reagent, Beckman Coulter, Fullerton, USA). Insulin concentrations were measured by enzyme-linked immunosorbent assay (Mercodia, Uppsala, Sweden).

Serum fatty acid composition in the triglyceride fraction was determined in pooled samples per group and per high-fat challenge at 0 h and 4 h and measured by gas-liquid chromatography as previously described [22].

#### Ex-vivo PBMC immune stimulation

PBMC immune response capacity in the fasted (0 h) condition and 4 h after the high-fat challenge was tested ex vivo and used as a measure of PBMC functionality. Ex vivo immune stimulation experiments were performed in a random subgroup of 13 lean and 15 obese subjects. Immediately after isolation PBMCs were re-suspended in RPMI 1640 culture medium with 10% heat-inactivated fetal calf serum and 1% penicillin and streptomycin. Cells in a concentration of 2.5×10^4^ per ml were stimulated for 2 h at 37°C with 1 ng/ml lipopolysaccharide (LPS). Subsequently, cells were centrifuged and supernatants collected and stored at −80°C until analysis. TNFα produced by the PBMCs was measured in the supernatants by enzyme-linked immunosorbent assay according to manufacturer’s instructions (R&D Systems Europe Ltd, Abingdon, United Kingdom). LPS-stimulated TNFα production was corrected for TNFα production in non-stimulated cells.

#### Body composition and abdominal fat distribution

Body weight was measured at screening and monitored during the study period. Body composition was determined on one study day by air-displacement plethysmography (BodPod; Life Measurement, Concord, CA) [25]. Abdominal fat distribution, i.e. abdominal visceral adipose tissue (VAT) and abdominal subcutaneous adipose tissue (SAT), was measured once during the study period using magnetic resonance imaging (MRI) in 17 lean, 18 obese and 4 obese diabetic subjects eligible to participate in this measurement. Axial T1-weighted spin echo images were acquired with a Philips Gyroscan NT Intera 1.0T scanner using the body coil, with the subjects in supine position. A total of 14 10-mm-thick slices with no intersection gap were acquired, with the first slice at the superior border of the vertebral body of L5, and the remaining slices superiorly. Images were acquired during breath-hold to avoid motion artefacts induced by breathing. Images were retrieved from the scanner using DICOM, and analysed using HIPPO (version 1.3), an IDL Virtual Machine 6.0-based freeware designed to quantify adipose tissue areas from MR images [26]. Automatically generated contour lines for SAT and VAT and the shape of Gaussian curve were manually adjusted by eye, as necessary. Retroperitoneal adipose tissue was excluded from VAT. VAT, SAT and VAT/SAT ratios derived from a single slice at the superior border of the vertebral body of L5 were used in the analysis.

### Statistical Analysis

The statistical packages PASW (version 17.0; SPSS Inc. Chicago IL) and SAS (version 9.1, SAS Institute Inc. 2004, Cary, NC, USA) were used for analysis. Differences in baseline characteristics between subject groups were analysed by analysis of variance (ANOVA) for single measures (i.e. age, body weight, body composition) or by linear mixed model for repeated measures (all other factors that were measured on all three consecutive study days) and followed by post-hoc LSD tests. Differences in responses for the different high-fat shakes and subject groups were analysed by linear mixed model. Delta values (changes from baseline) were used as dependent variables in the analysis and baseline values were included as covariables and time and shake were included as repeated factors in the model. If statistical significance was found, post-hoc LSD tests were performed to identify differences between shakes or groups. The number of obese diabetic subjects included in our trial was lower than intended, which may influence the chance of finding significant differences between subject groups. Therefore all statistical analyses were also performed without including the obese diabetic subject group. If outcomes differed between both analyses this is marked in the tables and indicated in the table footnotes.

The effect of VAT, SAT and VAT/SAT ratio on baseline and postprandial measures was only investigated within subject groups because of expected large differences in VAT and SAT between subject groups due to the predefined BMI categories. Obese diabetic subjects were excluded for this analysis due to the low number of subjects that was measured in the MRI scanner. For the analysis of the effect of body fat distribution VAT, SAT and VAT/SAT ratio were included as continuous covariables in the linear mixed model.

## Results

### Subject Baseline Characteristics

**Figure 1:**
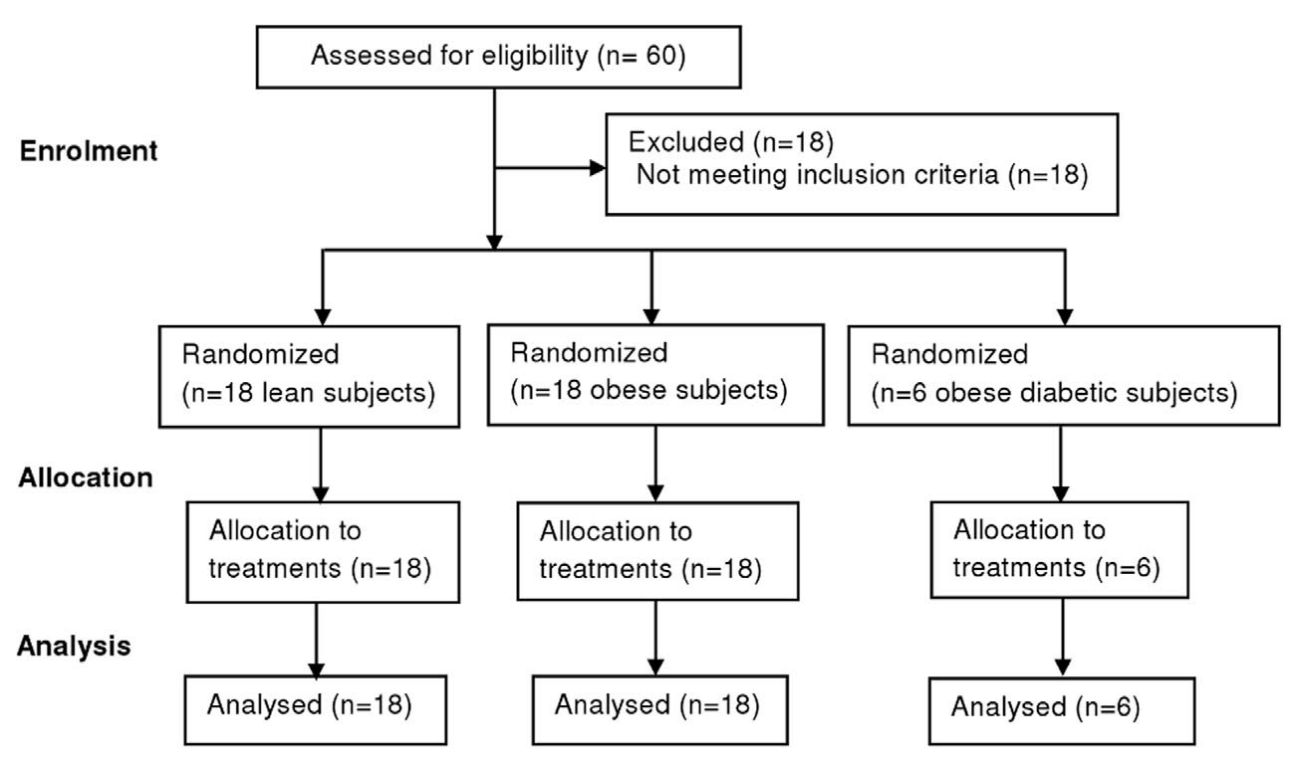
CONSORT flow diagram.

Between September 2009 and December 2009 42 men (18 lean subjects, 18 obese subjects and 6 obese diabetic subjects) were recruited and enrolled in the trial (figure 1). Baseline subject characteristics are displayed in table 2. BMI, bodyfat percentage, abdominal VAT, abdominal SAT, plasma fasting TAG and insulin concentrations were significantly higher in obese and obese diabetic subjects compared with lean subjects. Fasting plasma glucose concentration was higher in obese diabetic subjects compared with lean and obese subjects.

**Table 2:** Baseline characteristics of the participants.

	Lean(n = 18)	Obese(n = 18)	Obese diabetic(n = 6)
Age (y)	61.8±5.9	62.6±3.2	64.2±4.6
BMI (kg m^−2^)	23.8±0.8	32.4±3.0^1^	33.5±3.3^1^
Body fat (%)	21.9±5.7	38.2±5.2^1^	39.9±5.1^1^
VAT (cm^2^)^2^	102±40	231±75^1^	215±31^1^
SAT (cm^2^)^2^	163±52	350±99^1^	383±42^1^
VAT/SAT ratio^2^	0.64±0.20	0.68±0.24	0.57±0.12
TAG (mmol/L)	1.5±0.5	2.1±0.9^2^	2.0±1.0^2^
FFA (mmol/L)	0.51±0.21	0.51±0.13	0.58±0.11
Insulin (mmol/L)	6.1±2.8	13.4±6.6^2^	13.3±7.8^2^
Glucose (mmol/L)	5.2±0.4	5.5±0.4	7.2±1.0^2^

Values are expressed as mean ± standard deviation. Abbreviations: Free fatty acids (FFA), Triglycerides (TAG), Subcutaneous Adipose Tissue (SAT), Visceral adipose tissue (VAT).

1 Significantly different (p<0.05) from lean subjects.

2 For VAT, SAT en VAT/SAT ratio n = 17 for lean subjects, n = 18 for obese subjects and n = 4 for obese diabetic subjects.

### Serum Fatty Acid Composition

Baseline values and changes in fatty acid composition of the TAG fraction in pooled serum samples 4 h after the high-fat challenge are shown in table S1. The percentage palmitic acid was 1.1-fold higher after the SFA challenge, the percentage oleic acid was 1.6-fold higher after the MUFA challenge and the percentage DHA was 9.5-fold higher after the n-3 PUFA challenge, reflecting the composition of the shakes.

### Primary Study Outcomes

#### PBMC gene expression

Gene expression levels at baseline (fasting state) were not different between the subject groups. Changes in expression of *PDK4, LDLr, IL8* and *MCP1* expression were depending on the type of fat in the challenge. The MUFA challenge induced a lower decrease in *PDK4* expression at 2 h (p<0.001) if compared with the other challenges. The MUFA and n-3 PUFA challenge induced a higher increase in *MCP1* and *IL8* expression at 4 h (p<0.05) compared with the SFA challenge.

The high-fat challenge altered expression of several metabolic genes and inflammation-related genes (table S2). Changes in expression of the metabolic genes *ABCA1* and *LDLr* and the inflammation-related genes *IL1b* and *MCP1* depended on whether the subjects were lean, obese or obese diabetic. Changes in expression of *ABCA1* and *LDLr* were less pronounced for obese and obese diabetic subjects (p<0.05) compared with lean subjects. Differences between groups in expression changes of *IL1b* and *MCP1* were most likely due to higher increases of expression of these genes in obese diabetic subjects compared with lean and obese subjects, since the effects were not significant when obese diabetic subjects were excluded from analysis.

There were no interaction effects between subject groups and shakes for the measured genes.

### Secondary Study Outcomes

#### Plasma cytokines

Fasting plasma IL1β concentration was higher in obese diabetic subjects compared to lean and obese subjects (table 3). Changes in plasma concentrations of IL1β varied according to the type of fat in the challenge and whether the subjects were lean, obese or obese diabetic. Plasma concentrations of TNFα after the challenge were different between groups, with lower TNFα concentrations in lean compared with obese and obese diabetic subjects (p<0.05). TNFα responses over time were not different between the groups. Changes in plasma IL1β concentration differed among subject groups, however, the group and shake effects for IL1β were probably mainly due to the deviating IL1β responses of some obese diabetic subjects, since the shake and shake*group effects were not significant when the obese diabetic subjects were excluded from the analysis.

**Table 3:** Changes (mean ± sd) in plasma cytokines and ex vivo LPS-stimulated TNFα production of PBMCs after high fat shake consumption.

		SFA shake			MUFA shake			n-3 PUFA shake			Main effects			Interaction effects		
		0 h	Δ 2 h	Δ 4 h	0 h	Δ 2 h	Δ 4 h	0 h	Δ 2 h	Δ 4 h	group	time	shake	group*time	group*shake	shake*time
Plasma																
IL1β(pg/mL)	Lean	0.75±0.42	0.02±0.19	0.00±0.16	0.68±0.30	0.05±0.10	0.00±0.16	0.69±0.27	0.02±0.15	0.02±0.16	0.512	0.740	0.036^1^	0.676	0.041^1^	0.784
	Obese	0.70±0.51	0.09±0.14	0.07±0.12	0.75±0.49	0.00±0.10	0.04±0.16	0.76±0.55	0.05±0.25	−0.01±0.10						
	Obesediabetic^2^	1.44±1.03	0.01±0.12	−0.06±0.32	1.29±0.97	0.12±0.48	0.17±0.34	1.60±0.93	−0.24±0.45	−0.07±0.58						
TNFα (pg/mL)	Lean	6.54±1.55	0.04±0.54	−0.14±0.64	6.43±1.48	−0.19±0.64	−0.20 ±0.54	6.73±1.76	−0.26 ±0.60	−0.11±1.00	0.004	0.280	0.993	0.369	0.536	0.295
	Obese	7.27±2.04	0.22±0.53	−0.02±0.60	7.31±1.68	0.14±0.55	0.07±0.67	7.58±1.81	−0.10±0.65	−0.09±0.86						
	Obesediabetic	7.93±1.35	0.14±0.33	−0.28±0.52	8.15±1.50	0.19±0.78	−0.17 ±0.41	7.57±1.54	0.21±0.66	0.29±1.11						
Ex vivo LPS stimulated^3^																
TNFα (pg/mL)	Lean	164±92		−26±72	138±95		−5±85	210±262		−20±64	0.063	<0.001	0.900	0.084	0.962	0.639
	Obese	141±86		−50±86	162±130		−50±137	170±113		−67±84						

1 no significant effect when obese diabetic subjects are excluded from analysis, ^2^baseline values significantly different from those of lean and obese subjects, ^3^This measurement was done in a subsample of 13 lean and 15 obese subjects.

#### PBMC immune response capacity

Fasting levels and changes in PBMC immune response capacity after the high-fat challenges, measured as ex vivo LPS-stimulated TNFα production of PBMCs, were neither depending on the type of fat in the challenge, nor depending on the metabolic risk profile of the subjects (table 3). However, TNFα production was significantly lower (P<0.001) 4 h after the high-fat challenge compared with the fasting state.

#### Plasma FFA, TAG, insulin and glucose responses

**Figure 2:**
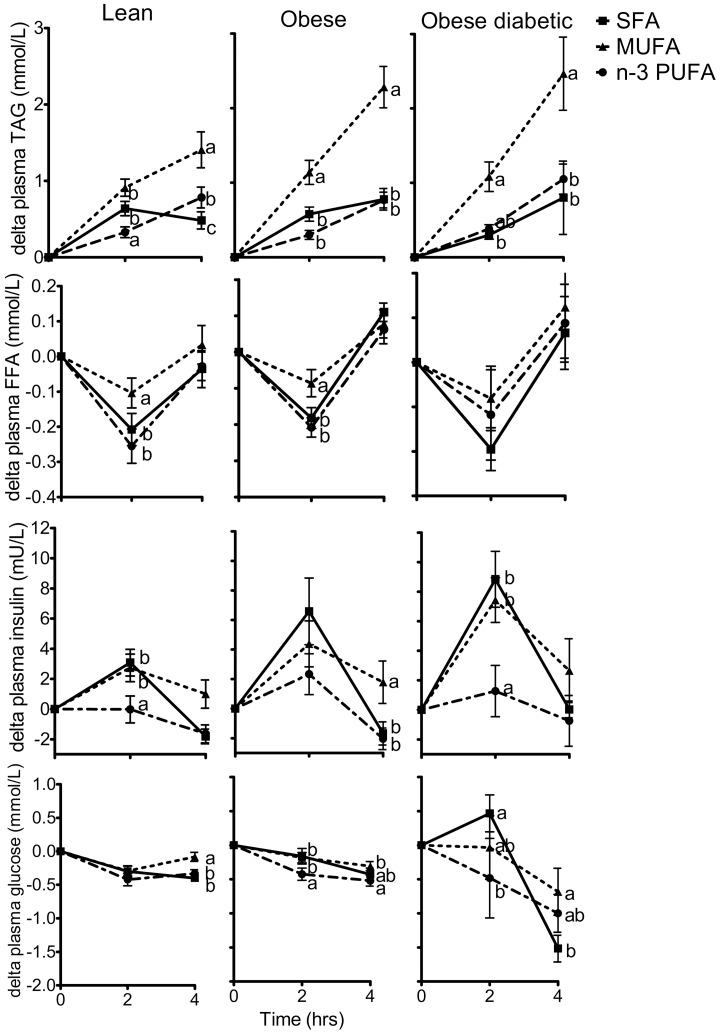
Changes in plasma metabolic parameters at 2 h and 4 h after high-fat shake consumption. Mean (± SEM) changes in plasma triglyceride (A), free fatty acid (B), insulin (C) and glucose (D) concentrations of lean subjects (n = 18), obese subjects (n = 18) and obese diabetic subjects (n = 6) after consumption of 3 different shakes, enriched in saturated fatty acids (SFA, line with squares), monounsaturated fatty acids (MUFA, dotted line with triangles) or n-3 polyunsaturated fatty acids (n-3 PUFA, dashed line with circles). Different letters indicate significant differences (p<0.05) between shakes at a given time.

Changes in FFA, TAG, insulin and glucose concentration after the different high-fat challenges in the subject groups are depicted in table 4 and figure 2. Concentrations of FFA, TAG, insulin and glucose were changed after all three high-fat challenges. The magnitude of the changes in these metabolites was depending on the type of fat consumed. The high MUFA challenge caused a higher total TAG response (p<0.001) and a reduced drop in FFA concentrations at 2 h (p<0.05) compared with the SFA and n-3 PUFA challenge. The n-3 PUFA challenge caused a less pronounced increase in insulin concentration (p<0.01) at 2 h compared with SFA and MUFA.

**Table 4:** Changes in metabolic parameters in plasma of lean subjects (n = 18), obese subjects (n = 18) and obese diabetic subjects (n = 6) at 2 h and 4 h after high fat shake consumption.

		SFA shake			MUFA shake			n-3 PUFA shake			Main effects			Interaction effects		
		0 h	Δ 2 h	Δ 4 h	0 h	Δ 2 h	Δ 4 h	0 h	Δ 2 h	Δ 4 h	group	time	shake	Group*time	Group*shake	Shake*time
TAG(mmol/L)	Lean	1.53±0.49	0.64±0.40	0.48±0.48	1.37±0.41	0.91±0.47	1.41±1.00	1.50±0.50	0.33±0.30	0.78±0.58	0.889	<0.001	<0.001	0.002	0.048	<0.001
	Obese	2.07±0.99	0.58±0.40	0.78±0.61	2.13±1.06	1.14±0.68	2.28±1.18	1.98±0.78	0.30±0.26	0.77±0.45						
	Obesediabetic	2.17±1.16	0.30±0.14	0.80±1.21	1.92±0.93	1.08±0.49	2.47±1.21	2.05±1.21	0.38±0.13	1.05±0.49						
FFA(mmol/L)	Lean	0.51±0.21	−0.21±0.19	−0.04±0.22	0.49±0.20	−0.10±0.18	0.03±0.23	0.55±0.23	−0.25±0.21	−0.03±0.17	0.004	<0.001	0.002	0.016	0.390	0.002
	Obese	0.48±0.10	−0.18±0.12	0.11±0.11	0.50±0.15	−0.09±0.16	0.08±0.16	0.54±0.15	−0.21±0.11	0.06±0.16						
	Obesediabetic	0.53±0.13	−0.20±0.12	0.07±0.20	0.58±0.13	−0.08±0.18	0.12±0.28	0.62±0.05	−0.12±0.25	0.09±0.21						
Insulin(mmol/L)	Lean	6.52±2.55	3.08±3.77	−1.82±2.08	5.80±3.38	2.73±3.88	1.00±3.98	6.02±2.55	−0.02±3.77	−1.64±2.56	0.017	<0.001	<0.001	0.028	0.808	<0.001
	Obese	13.52±7.40	6.59±9.60	−1.67±3.33	12.81±5.24	4.39±6.62	1.79±6.07	13.91±7.25	2.33±5.87	−2.04±3.09						
	Obesediabetic	12.66±7.50	8.85±4.64	0.03±1.40	13.57±8.78	7.43±3.71	2.66±5.30	13.79±8.49	1.27±4.26	−0.75±4.20						
Glucose(mmol/L)	Lean	5.19±0.39	−0.30±0.35	−0.40±0.21	5.17±0.35	−0.29±0.31	−0.08±0.28	5.29±0.53	−0.42±0.39	−0.33±0.25	0.872	<0.001	0.002	<0.001	0.660	<0.001
	Obese	5.50±0.41	−0.16±0.48	−0.43±0.39	5.53±0.43	−0.18±0.32	−0.31±0.28	5.54±0.28	−0.43±0.38	−0.52±0.33						
	Obesediabetic	7.22±0.94	0.47±0.67	−1.52±0.48	7.17±1.04	−0.03±1.14	−0.68±0.85	7.23±1.25	−0.48±1.43	−1.00±0.68						

Values are expressed as mean ± SD. Saturated fatty acid (SFA), monounsaturated fatty acid (MUFA), polyunsaturated fatty acid (PUFA), Free fatty acids (FFA), Triglycerides (TAG).

The changes in TAG, FFA, insulin and glucose concentrations were also different between lean, obese and obese diabetic subjects. For TAG response an interaction effect between group and shake (p = 0.048) was observed, meaning that the postprandial TAG response was different for lean, obese and obese diabetic subjects and depending on the type of high-fat shake consumed.

#### Abdominal fat distribution

Within the group of lean subjects and within the group of obese subjects there was no significant influence of abdominal fat distribution (i.e. VAT, SAT or VAT/SAT) on changes in plasma FFA, TAG, insulin or glucose concentrations, changes in PBMC gene expression, or changes in cytokine concentrations in response to the high-fat challenges.

## Discussion

This study shows that in response to a high-fat challenge, the expression of the metabolic genes *ABCA1, LDLr* and inflammatory genes *IL1β* and *MCP1* differed between lean, obese and obese subjects with diabetes. Moreover, plasma TAG, FFA, insulin and glucose concentrations after the challenge differed between subjects with distinct metabolic phenotypes. Comparison of responses to high-fat challenges high in SFA, MUFA or n-3 PUFA showed that high MUFA induced the most pronounced response, especially for TAG.

The decrease in *ABCA1* and the increase in *LDLr* expression in lean subjects in response to the challenge were comparable with observations in lean subjects in earlier studies performed by us and others [18], [27]. The novel observation of a less pronounced change in expression of these cholesterol metabolism genes in obese and obese diabetic subjects may reflect a less optimal metabolic adaptation response of their PBMCs to the challenge. This might be caused by lower lipid uptake by the cells and/or lower activation of gene expression in response to high-fat intake, possibly due to lowered sensitivity of the cells to lipids. Changes in expression of *ABCA1* and *LDLr* in response to a high-fat challenge might thus be considered as potential markers of health status.

The change in expression of the inflammatory genes *IL1β* and *MCP1* in response to the challenge was also different between groups, mainly due to larger increases in obese diabetic subjects, and not in obese subjects. We might consider these gene expression changes as indicators of a more severe metabolic phenotype; however we should interpret the study outcomes for the diabetic group with caution, since only a low number of diabetic subjects were included in the study. The greater change in IL1β expression in obese diabetic subjects was not reflected in plasma IL1β concentrations. This could be explained by the fact that IL1β is also produced by other cells than PBMCs. The low power to detect significant effects in the small obese diabetic subject group with high variation in cytokine response may also play a role.

PBMC gene expression responses to the challenges were also depending on the type of fat consumed. The n-3 PUFA and MUFA challenge induced higher increases in *MCP1* and *IL8* expression compared to the SFA challenge. This is in line with findings from a previous study showing that acute high n-3 PUFA intake induced a pro-inflammatory PBMC gene expression profile [18]. A possible explanation for this induction is that unsaturated fatty acids are more prone to oxidation than SFA and might induce more oxidative stress and in turn affect inflammatory status. However, no differences in expression changes of hypoxia inducible factor (*HIF1*α) were seen among the fat challenges (unpublished data). Another explanation may be that palmitic acid, which is generally more regularly consumed, poses less stress to PBMCs resulting in lower expression changes of inflammation genes than high doses of oleic acid or DHA. A high-fat palm oil shake may thus be less suitable to acutely challenge the system compared to shakes containing unsaturated fats.

After all high-fat challenges PBMC immune response capacity was reduced, but whether this reduction is an indication of lower inflammatory status or diminished cell immune functioning cannot be distinguished. Moreover, an effect of circadian rhythm on PBMCs cannot be excluded. Since PBMC immune response capacity was not different between subject groups, this ex vivo test after a challenge may not be a sensitive indicator for differences in health status.

The secondary study outcomes, plasma TAG, FFA, insulin and glucose concentrations after the challenge, clearly differed between subjects with distinct metabolic phenotypes. The differential changes in TAG and insulin concentrations in lean, obese and obese diabetic subjects are in line with results from postprandial studies showing higher TAG and insulin responses in obese and diabetic subjects after high-fat consumption [4], [7], [8], [28], [29]. We consider the differential changes in TAG and insulin in obese and obese diabetic subjects as a reflection of reduced cellular adaptation capacity to respond to a high-fat challenge.

The plasma metabolic responses to the challenges were also depending on the type of fat consumed. Comparison of responses to high in SFA, MUFA or n-3 PUFA challenges showed that high MUFA induced the most pronounced response, especially for TAG. The TAG-raising effect after a MUFA challenge was more pronounced in obese and obese diabetic subjects, suggesting that acute high MUFA intake might be a stronger metabolic challenge for subjects with a metabolic risk phenotype. It should be further investigated whether the plasma TAG response to a MUFA challenge could be used as a marker to detect small differences in health status in subjects with more comparable phenotypes.

The MUFA challenge may have been the most challenging for the biological system because it contained almost exclusively oleic acid (83% of total fat) while the other shakes contained a mixture of fatty acids. Our test shakes were not reflecting habitual meal intake, but used as a metabolic challenge test. However, because habitual intake of fish n-3 PUFA is very low we prepared an n-3 PUFA shake consisting of a mixture of palm oil and 55 gr fish oil, a well-tolerated dose in a former study at our group [18]. In the latter study, consumption of an n-3 PUFA shake containing this high dose of 55 gr n-3 PUFA and no palm oil, decreased expression of LXR signalling genes in PBMCs of young, lean men when compared to consumption of an SFA shake with 55 gr butter fat. Although we gave a higher total fat load, we observed no expression differences for these genes. This may be due to differences in fat types (palm oil vs. butter), shake composition, sampling time (4 h vs 6 h) or age of the subjects.

Remarkably, our n-3 PUFA shake induced a less pronounced increase in insulin concentration compared with the other shakes. Only few other studies have reported acute insulin-lowering effects of n-3 PUFA intake [15], [30]. Peak insulin concentrations are reached normally between 0–2 h after a meal [4], [19], [31], but we lack data for these early time points. n-3 PUFA intake might have caused an earlier insulin peak resulting in lower insulin concentrations at later time points.

The amount of VAT, SAT or the VAT/SAT ratio did not influence the response to a high-fat challenge within subject groups. Although the number of subjects per group might have been too small to detect a significant effect of fat distribution, it is also arguable whether high VAT is strongly affecting metabolic health; some studies did find an effect of VAT, mainly on TAG response [9], [32], [33], but others suggested that liver fat might be a more sensitive determinant of metabolic health [34], [35]. VAT comprises only about 10% of total body fat, which might also be a reason that no VAT-dependent effect was seen.

In our study we monitored different aspects of the response to high-fat challenges, i.e. plasma metabolites, inflammatory proteins, PBMC gene expression and immune cell functioning. We were able to detect differential changes in expression of metabolic parameters and certain genes between the selected subject groups differing in BMI and health status. However, for identification of small differences between responses of subjects that are phenotypically more similar, more sensitive monitoring of the response is needed. Whole genome transcriptome, proteome or metabolome profiling tools could be used for more extensive characterization of the challenge response. As shown in previous studies, extensive profiling could identify subtle changes of genes, proteins or metabolites in pathways and clusters [14], [36]. The combination of challenge tests and extensive profiling may reveal changes in health status at a very early stage [37].

In conclusion, of the three fat types studied most pronounced changes were seen for the high oleic sunflower oil (MUFA). Therefore this fat type seems the most promising challenge to test metabolic response capacity. We identified several genes expressed in PBMCs and plasma metabolic measures that were differently responding to a high-fat challenge in subjects with distinct metabolic risk phenotypes. These potential markers are likely candidates to be further tested and used in high-fat challenge tests to define metabolic response capacity of subjects.

## Supporting Information

Table S1 **Serum fatty acid composition of the TAG fraction (% of total) of pooled samples for group and time point.**
(DOC)

Table S2 **Changes in PBMC gene expression of lean subjects (n = 18), obese subjects (n = 18) and obese diabetic subjects (n = 6) at 2 h and 4 h after high-fat shake.**
(DOC)

Checklist S1 **CONSORT checklist.**
(DOC)

Protocol S1 **Trial protocol.**
(DOC)
