# Supplementary material for: Responses to High-Fat Challenges Varying in Fat Type in Subjects with Different Metabolic Risk Phenotypes: A Randomized Trial
Source: PLoS One. 2012 Jul 23;7(7):e41388. doi: 10.1371/journal.pone.0041388 (PMC3402390; doi:10.1371/journal.pone.0041388)
Supplement: Table S1 — Serum fatty acid composition of the TAG fraction (% of total) of pooled samples for group and time point. (DOC) [file pone.0041388.s001.doc]

Table S1 Serum fatty acid composition of the TAG fraction (% of total) of pooled samples for group and time point. Only values for palmitic acid, oleic acid and DHA are displayed

|  | SFA shake | | MUFA shake | | n-3 PUFA shake | |
| --- | --- | --- | --- | --- | --- | --- |
|  | Baseline | Δ 4 hrs | Baseline | Δ 4 hrs | Baseline | Δ 4 hrs |
| **Palmitic acid** |  |  |  |  |  |  |
| Lean | 27.45 | 2.87 | 25.76 | -11.54 | 27.06 | -0.56 |
| Obese | 28.14 | 2.54 | 27.89 | -11.22 | 29.13 | 0.30 |
| Obese diabetic | 27.77 | 3.74 | 26.99 | -11.09 | 28.74 | 0.32 |
| **Oleic acid** |  |  |  |  |  |  |
| Lean | 35.78 | 2.52 | 38.14 | 22.79 | 37.41 | -2.48 |
| Obese | 36.14 | 1.12 | 36.02 | 20.68 | 35.71 | -0.55 |
| Obese diabetic | 36.79 | 0.72 | 39.02 | 20.21 | 36.73 | -1.48 |
| **DHA** |  |  |  |  |  |  |
| Lean | 0.68 | -0.09 | 0.44 | -0.12 | 0.32 | 4.56 |
| Obese | 0.66 | -0.09 | 0.63 | -0.16 | 0.55 | 2.86 |
| Obese diabetic | 0.96 | 0.27 | 0.90 | -0.39 | 0.54 | 3.31 |
