# Supplementary material for: Responses to High-Fat Challenges Varying in Fat Type in Subjects with Different Metabolic Risk Phenotypes: A Randomized Trial
Source: PLoS One. 2012 Jul 23;7(7):e41388. doi: 10.1371/journal.pone.0041388 (PMC3402390; doi:10.1371/journal.pone.0041388)
Supplement: Table S2 — Changes in PBMC gene expression of lean subjects (n = 18), obese subjects (n = 18) and obese diabetic subjects (n = 6) at 2 h and 4 h after high-fat shake. (DOC) [file pone.0041388.s002.doc]

Table S2 Changes in PBMC gene expression of lean subjects (n=18), obese subjects (n=18) and obese diabetic subjects (n=6) at 2 h and 4 h after high-fat shake

|  |  | SFA shake | | MUFA shake | | n-3 PUFA shake | | Main effects | | | Interaction effects | | |
| --- | --- | --- | --- | --- | --- | --- | --- | --- | --- | --- | --- | --- | --- |
|  |  | Δ 2 h | Δ 4 h | Δ 2 h | Δ 4 h | Δ 2 h | Δ 4 h | group | time | shake | group* time | group*shake | shake*time |
| Metabolism | |  |  |  |  |  |  |  |  |  |  |  |  |
| ABCA1 | Lean | 0.49± 1.03 | 1.05± 0.78 | 0.36± 0.62 | 0.59± 0.70 | 0.42± 0.89 | 0.39± 0.70 | 0.007 | <0.001 | 0.235 | 0.038 | 0.877 | 0.261 |
|  | Obese | 0.42± 0.84 | 0.28± 0.93 | 0.42± 0.84 | 0.36± 1.11 | 0.03± 0.55 | 0.03± 0.62 |  |  |  |  |  |  |
|  | Obese diabetic | -0.11±0.75 | 0.34± 0.77 | 0.31± 0.70 | 0.10± 0.75 | 0.36± 0.97 | 0.41± 1.04 |  |  |  |  |  |  |
| CYP27A1 | Lean | 0.41± 0.60 | 0.53± 0.55 | 0.41± 0.63 | 0.66± 0.49 | 0.39± 0.43 | 0.65± 0.65 | 0.576 | <0.001 | 0.682 | 0.325 | 0.704 | 0.921 |
|  | Obese | 0.40± 0.53 | 0.34± 0.64 | 0.47± 0.72 | 0.60± 1.07 | 0.25± 0.63 | 0.32± 0.58 |  |  |  |  |  |  |
|  | Obese diabetic | 0.10± 0.53 | 0.44± 0.75 | 0.14± 0.50 | 0.21± 0.55 | 0.53± 0.44 | 0.50± 0.46 |  |  |  |  |  |  |
| LDLr | Lean | -0.45±0.49 | -0.40±0.58 | -0.67±0.59 | -0.19±0.57 | -0.51±0.46 | -0.42±0.41 | 0.034^1^ | <0.001 | 0.040^1^ | <0.001 | 0.642 | 0.025 |
|  | Obese | -0.28±0.46 | -0.16±0.52 | -0.28±0.58 | 0.03± 0.80 | -0.31±0.48 | -0.24±0.59 |  |  |  |  |  |  |
|  | Obese diabetic | -0.16±0.33 | 0.01± 0.56 | -0.11±0.45 | 0.33± 0.40 | 0.17± 0.46 | 0.33±0.49 |  |  |  |  |  |  |
| LXRα | Lean | 0.06± 0.61 | 0.21± 0.73 | 0.04± 0.73 | 0.05± 0.66 | -0.02±0.48 | -0.16±0.59 | 0.225 | 0.789 | 0.904 | 0.620 | 0.937 | 0.108 |
|  | Obese | -0.05±0.74 | 0.12± 0.72 | -0.23±0.81 | -0.25±0.81 | 0.05± 0.39 | -0.02±0.40 |  |  |  |  |  |  |
|  | Obese diabetic | -0.25±0.38 | 0.20± 0.74 | 0.26± 0.33 | 0.10± 0.55 | 0.48± 0.86 | 0.13± 0.87 |  |  |  |  |  |  |
| PDK4 | Lean | -0.28±0.64 | 0.39± 0.69 | 0.00± 0.53 | 0.53± 0.60 | -0.55±0.43 | 0.46± 0.60 | 0.563 | <0.001 | <0.001 | 0.292 | 0.770 | <0.001 |
|  | Obese | -0.28±0.61 | 0.25± 0.65 | 0.06± 0.37 | 0.55± 0.46 | -0.47±0.31 | 0.29± 0.64 |  |  |  |  |  |  |
|  | Obese diabetic | -0.22±0.50 | 0.42± 0.64 | 0.58± 1.55 | 0.88± 1.63 | -0.38±0.50 | 0.28± 0.39 |  |  |  |  |  |  |
| SREBP1 | Lean | -0.05±0.54 | 0.13±0.38 | 0.16±0.53 | 0.32±0.51 | 0.17±0.48 | 0.05±0.69 | 0.006^1^ | 0.343 | 0.879 | 0.338 | 0.638 | 0.379 |
|  | Obese | -0.08±0.62 | -0.06±0.62 | 0.16±0.81 | 0.12±0.76 | 0.06±0.31 | -0.03±0.43 |  |  |  |  |  |  |
|  | Obese diabetic | -0.11±0.37 | 0.02±0.63 | -0.30±0.58 | 0.04±0.84 | -0.06±0.59 | 0.12±0.53 |  |  |  |  |  |  |
| Inflammation | |  |  |  |  |  |  |  |  |  |  |  |  |
| IL1b | Lean | 0.13± 0.64 | 0.02± 0.62 | 0.07± 0.78 | 0.41± 0.87 | -0.08±0.37 | 0.07± 0.85 | 0.351 | <0.001^1^ | 0.293^2^ | 0.014^1^ | 0.638 | 0.167 |
|  | Obese | -0.13±1.16 | -0.11±0.65 | 0.08± 0.71 | 0.18± 1.10 | -0.11±0.60 | 0.23± 0.56 |  |  |  |  |  |  |
|  | Obese diabetic | 0.35± 0.49 | 0.54± 0.85 | 0.61± 1.73 | 1.17± 1.99 | 0.18± 0.72 | 0.79± 1.04 |  |  |  |  |  |  |
| IL8 | Lean | 0.20± 1.46 | 0.98± 1.28 | 0.10± 1.16 | 1.89± 1.35 | -0.35±1.15 | 1.68± 1.83 | 0.359 | <0.001 | 0.032 | 0.239 | 0.932 | 0.003 |
|  | Obese | -0.86±2.09 | 0.68± 1.38 | 0.18± 1.44 | 1.59± 1.67 | -0.66±1.18 | 2.01± 1.36 |  |  |  |  |  |  |
|  | Obese diabetic | 0.02± 1.00 | 1.74± 0.70 | 0.27± 1.42 | 2.04± 1.29 | 0.13± 2.68 | 2.48± 1.84 |  |  |  |  |  |  |
| MCP1 | Lean | 0.48± 1.11 | 0.31± 0.91 | 0.37± 1.49 | 1.37± 1.51 | 0.39± 0.92 | 0.94± 1.36 | 0.500 | <0.001 | 0.006 | 0.037^1^ | 0.730 | 0.015 |
|  | Obese | -0.02±1.05 | 0.22± 1.40 | 0.36± 1.51 | 0.97± 1.89 | 0.29± 0.88 | 0.77± 0.69 |  |  |  |  |  |  |
|  | Obese diabetic | 0.65± 1.22 | 1.11± 1.08 | 1.22± 0.69 | 1.96± 0.93 | 0.75± 1.55 | 1.77± 1.67 |  |  |  |  |  |  |
| NFκB1 | Lean | 0.09±0.78 | 0.21± 0.51 | -0.13±0.46 | -0.16±0.85 | 0.07±0.47 | -0.07±0.56 | 0.113 | 0.927 | 0.870 | 0.808 | 0.278 | 0.877 |
|  | Obese | -0.04±0.61 | -0.28±0.59 | 0.02±0.72 | -0.16±0.87 | -0.03±0.37 | 0.10±0.69 |  |  |  |  |  |  |
|  | Obese diabetic | 0.03±0.39 | 0.08±0.47 | 0.14±0.50 | 0.10± 0.58 | -0.05±0.99 | 0.08±0.64 |  |  |  |  |  |  |
| TNFα | Lean | 0.40± 0.55 | 0.33± 0.44 | 0.45± 0.65 | 0.27± 0.77 | 0.26± 0.42 | 0.13± 0.47 | 0.761 | <0.001 | 0.085 | 0.280 | 0.750 | 0.529 |
|  | Obese | 0.19± 0.57 | 0.06± 0.40 | 0.38± 0.69 | 0.25± 0.84 | 0.33± 0.50 | 0.37± 0.55 |  |  |  |  |  |  |
|  | Obese diabetic | 0.51± 0.52 | 0.61± 0.45 | 0.48± 0.49 | 0.57± 0.50 | -0.09±0.55 | 0.09± 0.51 |  |  |  |  |  |  |

Data are expressed as mean changes ±SD. ^1^no significant effect when obese diabetic subjects were excluded from analysis, ^2^significant effect (p 0.034) when obese diabetic subjects were excluded from analysis

Saturated fatty acid (SFA), monounsaturated fatty acid (MUFA), polyunsaturated fatty acid (PUFA). ATP-binding cassette, sub-family A member 1 (ABCA1), cytochrome P450, family 27, subfamily A, polypeptide 1 (CYP27A1), low density lipoprotein receptor (LDLr), liver X receptor (LXR), Monocyte chemotactic protein 1 (MCP1), nuclear factor of kappa light polypeptide gene enhancer in B-cells 1 (NFκB1), pyruvate dehydrogenase kinase, isozyme 4 (PDK4), Saturated fatty acids (SFA), sterol regulatory element binding transcription factor 1 (SREBP1).
