# Supplementary material for: Responses to High-Fat Challenges Varying in Fat Type in Subjects with Different Metabolic Risk Phenotypes: A Randomized Trial
Source: PLoS One. 2012 Jul 23;7(7):e41388. doi: 10.1371/journal.pone.0041388 (PMC3402390; doi:10.1371/journal.pone.0041388)
Supplement: Protocol S1 — Trial protocol. (DOC) [file pone.0041388.s004.doc]

**Protocol S1**

**Effects of fatty acids on postprandial inflammatory response of healthy obese and type 2 diabetic obese subjects**

Susan J van Dijk, MSc

Lydia A. Afman, PhD

Marco R. Mensink, PhD, MD

Edith JM Feskens, PhD

Michael Müller, PhD

Nutrition, Metabolism and Genomics Group

Division of Human Nutrition

Wageningen University

Bomenweg 2

6703 HD Wageningen

The Netherlands

e-mail: Susan.vanDijk@wur.nl

Phone: +31 (0)317-48 57 91

Fax: +31 (0)317-48 33 42

Effects of fatty acids on postprandial inflammatory response of healthy obese and type 2 diabetic obese subjects

| Protocol ID | 28001 |
| --- | --- |
| Short title | PIFA study |
| Version | 2 |
| Date | 12-06-09 |
| Principal Investigator | Lydia A. Afman, PhD  Nutrition, Metabolism and Genomics Group  Division of Human Nutrition  e-mail: Lydia.Afman@wur.nl  phone:+ 31(0)317-485789  fax: +31(0)317-483342 |
| Investigator 2: | Susan J van Dijk, MSc  Nutrition, Metabolism and Genomics Group  Division of Human Nutrition  e-mail: Susan.vanDijk@wur.nl  phone:+ 31(0)317-485791  Fax: +31 (0)317-483342 |
| Sponsor | Dutch Diabetes Research Foundation |
| Independent physician(s) | Dr. J.J. van Binsbergen |
| Laboratory sites | Division of Human Nutrition  Wageningen University  Bomenweg 2  6703 HD Wageningen  The Netherlands |

**PROTOCOL SIGNATURE SHEET**

| **Name** | **Signature** | **Date** |
| --- | --- | --- |
| **Project leader:**  Prof.dr. M. Müller  Chair in Nutrition, Metabolism and Genomics |  |  |
| **Principal investigator:**  Dr.ir. L.A. Afman  Assistant Professor |  |  |
| **Investigator 2:**  Ir. SJ van Dijk  PhD student |  |  |

**TABLE OF CONTENTS**

[LIST OF ABBREVIATIONS AND RELEVANT DEFINITIONS 5](#__RefHeading___Toc228585893)

[SUMMARY 6](#__RefHeading___Toc228585894)

[1. INTRODUCTION AND RATIONALE 8](#__RefHeading___Toc228585895)

[1.1 Scientific evidence 8](#__RefHeading___Toc228585896)

[1.2 Study aim: 9](#__RefHeading___Toc228585897)

[2. OBJECTIVES 10](#__RefHeading___Toc228585898)

[2.1 Research questions and hypotheses 10](#__RefHeading___Toc228585899)

[3. STUDY DESIGN 11](#__RefHeading___Toc228585900)

[3.1 Intervention study 11](#__RefHeading___Toc228585901)

[4. STUDY POPULATION 12](#__RefHeading___Toc228585902)

[4.1 Population 12](#__RefHeading___Toc228585903)

[4.2 Screening visit 12](#__RefHeading___Toc228585907)

[4.3 Inclusion criteria: 12](#__RefHeading___Toc228585908)

[4.4 Exclusion criteria 13](#__RefHeading___Toc228585911)

[4.5 Sample size calculation 14](#__RefHeading___Toc228585913)

[5. INTERVENTION 15](#__RefHeading___Toc228585914)

[5.1 Intervention meals 15](#__RefHeading___Toc228585915)

[5.2 Guidelines during the study period 15](#__RefHeading___Toc228585916)

[6. METHODS 17](#__RefHeading___Toc228585917)

[6.1 Study outcomes 17](#__RefHeading___Toc228585918)

[6.1.1 Measurements of primary study outcomes 17](#__RefHeading___Toc228585919)

[6.1.2 Measurements of secondary study outcomes 17](#__RefHeading___Toc228585920)

[6.1.3 Measurements of tertiary study outcomes 18](#__RefHeading___Toc228585922)

[6.2 Randomisation and blinding 18](#__RefHeading___Toc228585926)

[6.2 Study procedures 18](#__RefHeading___Toc228585927)

[6.2.1 Information meeting 19](#__RefHeading___Toc228585928)

[6.2.2 Screening 19](#__RefHeading___Toc228585929)

[6.2.3 Preparations prior to the Intervention 19](#__RefHeading___Toc228585930)

[6.2.4 Intervention 19](#__RefHeading___Toc228585932)

[6.3 Withdrawal of individual participants 21](#__RefHeading___Toc228585935)

[6.4 Follow-up of participants after withdrawal 21](#__RefHeading___Toc228585936)

[6.5 Premature termination of the study 21](#__RefHeading___Toc228585937)

[7. SAFETY REPORTING 22](#__RefHeading___Toc228585938)

[7.1 Section 10 WMO event 22](#__RefHeading___Toc228585939)

[7.2 Adverse and serious adverse events 22](#__RefHeading___Toc228585940)

[7.3 Follow-up of adverse events 22](#__RefHeading___Toc228585941)

[8. STATISTICAL ANALYSIS 23](#__RefHeading___Toc228585942)

[9. ETHICAL CONSIDERATIONS 25](#__RefHeading___Toc228585947)

[9.1 Regulation statement 25](#__RefHeading___Toc228585948)

[9.2 Recruitment and consent 25](#__RefHeading___Toc228585949)

[9.3 Privacy 25](#__RefHeading___Toc228585950)

[9.4 Benefits and risks assessment, group relatedness 25](#__RefHeading___Toc228585951)

[9.4.1 Potential value of the research 25](#__RefHeading___Toc228585952)

[9.4.2 Benefits for the participants 26](#__RefHeading___Toc228585954)

[9.4.3 Risks for the participants 26](#__RefHeading___Toc228585955)

[9.5 Compensation for injury 27](#__RefHeading___Toc228585956)

[10. ADMINISTRATIVE ASPECTS AND PUBLICATION 29](#__RefHeading___Toc228585957)

[10.1 Handling and storage of data and documents 29](#__RefHeading___Toc228585958)

[10.2 Amendments 29](#__RefHeading___Toc228585959)

[10.3 End of study report 29](#__RefHeading___Toc228585960)

[10.4 Public disclosure and publication policy 29](#__RefHeading___Toc228585961)

[REFERENCES 30](#__RefHeading___Toc228585962)

# LIST OF ABBREVIATIONS AND RELEVANT DEFINITIONS

| AE | Adverse Event |
| --- | --- |
| AR | Adverse Reaction |
| CCMO | Central Committee on Research Involving Human Subjects |
| DHA | Docosahexaenoic acid |
| EPA | Eicosapentaenoic acid |
| (F)FA | (Free) Fatty acids |
| IC | Informed Consent |
| IMP | Investigational Medicinal Product |
| Hb | Hemoglobin |
| LPS | Lipopolysaccharide |
| METC | Medical research ethics committee (MREC); in Dutch: medisch ethische toetsing commissie (METC) |
| MRI | Magnetic Resonance Imaging |
| MUFA | Mono-unsaturated fatty acids |
| OGTT | Oral glucose tolerance test |
| PBMC | Peripheral blood mononuclear cells |
| PUFA | Poly-unsaturated fatty acids |
| PWA | Pulse wave analysis |
| Q-PCR | Quantitative real time-polymerase chain reaction |
| (S)AE | Serious Adverse Event |
| SFA | Saturated fatty acids |
| Sponsor | The sponsor is the party that commissions the organization or performance of the research, for example a pharmaceutical  company, academic hospital, scientific organization or investigator. A party that provides funding for a study but does not commission it is not regarded as the sponsor, but referred to as a subsidizing party. |
| SEVR | Subendocardial Viability Ratio |
| TG | Triglyceride |
| WMO | Medical Research Involving Human Subjects Act (Wet Medisch-wetenschappelijk Onderzoek met Mensen) |

# SUMMARY

**Rationale:**

Consumption of high-fat diets can lead to postprandial dyslipidemia, impairment of endothelial function, activation of immune cells and changes in gene expression profiles of immune cells such as peripheral blood mononuclear cells (PBMC).

Recently it was shown that postprandial gene expression profiles of PBMC and plasma triglyceride (TG) and free fatty acid (FFA) responses are dependent on the type of dietary fat consumed (i.e. saturated, monounsaturated and polyunsaturated). Since obese and diabetic subjects usually are in a pro-inflammatory state and have dyslipidemia and endothelial dysfunction we are interested in the effect of different fatty acids in a high load on the PBMC gene expression profiles, plasma cytokine profiles and endothelial function of these subjects.

**Objectives**:

The main objective is to elucidate the acute effects of an oral intake of either saturated, monounsaturated or polyunsaturated fatty acids on PBMC whole genome expression of obese and type 2 diabetic obese subjects.

Secondary objectives are to elucidate the acute effects of the fat loads on PBMC inflammatory response capacity and endothelial function of these subjects. Tertiary objectives are to investigate the effects of the fat loads in the groups of subjects on plasma lipid profiles, plasma glucose and insulin and on monocyte and lymphocyte whole genome expression.

**Study design and study population:**

A single-blind cross-over dietary intervention with subjects (male, 50-70 yrs) with different risk profiles. Twenty-two obese type 2 diabetic subjects will be studied and compared to age and BMI matched healthy obese controls and to age-matched healthy, lean controls.

**Intervention**:

Each participant will consume a high SFA milkshake, a high MUFA milkshake and a high PUFA milkshake after overnight fasting.

**Study outcome:**

The main study outcome is change in PBMC whole genome expression. Secondary study outcomes are changes in inflammatory response capacity and endothelial function. Other study outcomes are plasma lipid profiles, plasma glucose and insulin values and monocyte and lymphocyte whole genome expression.

**Nature and extent of the burden and risks associated with participation, benefit and group relatedness:**

During a screening visit 3 ml blood will be drawn and urine will be collected after a overnight fast and body weight, length, waist and blood pressure will be measured. An oral glucose tolerance test will be performed. A general and medical questionnaire will be used.

During the study period of 8 weeks each participant will visit the university on 3 mornings, separated by at least one week, and will consume within 15 minutes a milkshake containing 95 grams of fat. Blood will be collected and endothelial function will be measured both before consuming the milkshakes (baseline, T=0) and 2 hrs (T=2) and 4 hrs (T=4) after consumption of the shakes. Blood will be drawn by normal venopuncture. At time points 0 and 2 hrs 60 ml blood will be collected and at time point 4 hr 52 ml will be collected, bringing it to a total of 172 ml per day. Hb values of each participant will be monitored during the study to be sure that blood collection will not lead to anaemia.

Participants have to collect a small amount of faeces at one time point during the study period. The body composition of the participants will be measured in the Bod Pod. In addition, the participants will visit the radiology department of the hospital Gelderse Vallei in Ede for a MRI scan to determine their abdominal bodyfat distribution.

The time investment requested from the participants is 1 hour at the information meeting, 2.5 hours at a screening session, 3 x 4.5 hours at the intervention days and 1 hour for the MRI scan. The risks associated with venous blood drawing by venopuncture are minimal. The consumption of the milkshakes is not expected to be associated with discomfort, but could, in rare cases, have adverse effects such as a mild gastrointestinal discomfort (fishy aftertaste in case of PUFA shake, belching, flatulence or loose stools). MRI is a safe procedure, with no known health risk as long as no contraindication is met (see paragraph 9.4.3). MRI can result in a ‘coincidence’ finding. These findings should be reported and subjects will be informed about this.

# INTRODUCTION AND RATIONALE

## 1.1 Scientific evidence

Consumption of a high-fat diet can elicit a postprandial inflammatory response in the human body. This response can be characterized by the activation of white blood cells, an increase in plasma acute phase proteins, impaired endothelial function and an elevation of plasma triglyceride (TG) and free fatty acid (FFA) levels (1-4).

PBMC are a subset of white blood cells that can be isolated from blood relatively easily. PBMC gene expression profiles can be used to distinguish a variety of diseases from the health state and they can reflect nutrition-related changes (5-7). Recently it was demonstrated that postprandial gene expression profiles of PBMC from healthy subjects are dependent on the type of dietary fat consumed (Bouwens et al., unpublished data). The differences in gene expression profiles were, beside other effects, characterized by differential effects on oxidative stress and inflammation.

The presence of obesity and type 2 diabetes are factors that can influence the postprandial inflammatory response (8). Obese subjects, compared to healthy lean subjects, have more pronounced postprandial inflammatory responses in addition to chronic low-grade inflammation in the fasting state (9). Subjects with type 2 diabetes show high postprandial inflammatory responses correlating with their degree of insulin resistance (10). Both groups of subjects display higher postprandial concentrations of TG, FFA and glucose, together with a delayed clearance, which can cause endothelial dysfunction and ultimately can lead to cardiovascular diseases.

Postprandial inflammatory response

The amount of saturated fatty acids and the amount of n-3 polyunsaturated fatty acids in a meal have emerged as important determinants of the magnitude of the postprandial response as measured by TG, FFA and glucose. However, the effects of different fatty acids on the postprandial inflammatory response in obese and type 2 diabetic subjects is not well known. Therefore we will investigate the effect of different types of dietary fatty acids (i.e. saturated, monounsaturated and polyunsaturated) on PBMC, monocyte and lymphocyte whole genome expression profiles, inflammatory response capacity, endothelial function, plasma lipid profiles and plasma glucose and insulin levels in obese and type 2 diabetic subjects that display different stages of postprandial dyslipidemia and endothelial dysfunction.

Studying the postprandial inflammatory response of these subjects will also provide us with more information about development of dyslipidemia and endothelial dysfunction and the influence of fat quality on this.

Phenotyping of the subjects

The phenotype of the subjects can be of importance for their inflammatory response capacity. We will measure some extra parameters for more comprehensive phenotyping of the subjects.

Not only the BMI of a subjects but also the body composition and the location of the fat are important determinants of health (11). Body composition will be measured with the use of the BodPod. Magnetic Resonance Imaging (MRI) will be used to measure abdominal fat distribution, i.e. intra-abdominal fat and subcutaneous abdominal fat.

The human gut flora is a complex microbial ecosystem, which also appears to be of key importance in health and disease (12). Therefore, faecal microflora profiling will be performed. Data on bacterial populations of the faecal flora of the subjects and data about body composition and body fat distribution will give more information about the metabolic phenotype of the subjects.

## 1.2 Study aim:

In the current study we aim to investigate the acute effects of an oral intake of different dietary fatty acids on PBMC gene expression profile in groups with a different risk profile (i.e. healthy lean, healthy obese and type 2 diabetic obese subjects). Three different fat loads of 95 g will be given to the subjects, using either saturated fatty acid (SFA), monounsaturated fatty acids (MUFA) or polyunsaturated fatty acids (PUFA) as basal constituents.

We also aim to investigate the effect of the different fatty acids in the risk groups on inflammatory response capacity, endothelial function, plasma lipid profiles, plasma glucose and insulin levels and monocyte and lymphocyte gene expression profiles.

# OBJECTIVES

The objectives are formulated to elucidate the effects of the different fatty acids in the high fat loads on gene expression of PBMC in type 2 diabetic subjects compared to healthy obese and healthy lean subjects. The main objective of this study is to elucidate whether the high fat loads (containing either SFA, MUFA or PUFA) will have different effects on PBMC gene expression in the three risk groups of subjects. Secondary objectives are to elucidate the effects of the fat loads in the different risk groups on inflammatory response capacity and endothelial function. Tertiary objectives are to investigate the effect of these fat loads in these subjects on plasma lipid profiles, glucose and insulin and monocyte and lymphocyte gene expression profiles. For more comprehensive phenotyping of the subjects we will also measure body composition, bodyfat distribution and fecal micro flora.

## 2.1 Research questions and hypotheses

1. Can intake of high fat loads with different fatty acids (SFA, MUFA, PUFA) result in differential changes in **PBMC gene expression** in the different groups of subjects?

🡪 *The PBMC gene expression profiles of the high risk subjects will be more affected compared to gene expression profiles of the healthy subjects.*

2. Can the **PBMC inflammatory response capacity** be influenced by fatty acid intake and will these changes be different for the high risk subjects?

*🡪 Obese and type 2 diabetic subjects will have an impaired inflammatory response capacity. The different fatty acids will differently influence this inflammatory response capacity*

3. Can intake of high fat loads with different fatty acids result in differential changes in **endothelial function** and will these changes be different for the high risk subjects?

🡪 *The different fatty acid compositions of the shakes will result in different changes in endothelial function. The high fat load will result in a higher degree of endothelial dysfunction in the high-risk subjects compared to the healthy subjects.*

# 3. STUDY DESIGN

## 3.1 Intervention study

This study is a single-blind cross-over intervention (challenge) study, planned to run from the end of October till half December 2009.

In this study obese type 2 diabetic subjects will be studied and compared to age and BMI matched healthy obese controls and to age-matched healthy lean controls.

A total of 22 type 2 diabetic obese men, 22 obese men and 22 lean men will consume high-fat milkshakes containing 95 g of fat, mainly consisting of either SFA, MUFA or PUFA. No differences can be seen or tasted between the shakes.

The 22 type 2 diabetic obese, 22 obese and 22 lean men will be randomly divided in 6 groups to determine in which order they will receive the milkshakes. The groups will receive a different high-fat shake every two weeks (see table 1). There will be a minimal period of one week between two testing days, in order to exclude carry over effects. Six persons will be evaluated on each testing day.

Table1: Experimental study design

|  |  | Week 1/2 | Week 3/4 | Week 5/6 |
| --- | --- | --- | --- | --- |
| Intervention order | Group 1 | SFA | MUFA | PUFA |
|  | Group 2 | SFA | PUFA | MUFA |
|  | Group 3 | MUFA | SFA | PUFA |
|  | Group 4 | MUFA | PUFA | SFA |
|  | Group 5 | PUFA | SFA | MUFA |
|  | Group 6 | PUFA | MUFA | SFA |

# 4. STUDY POPULATION

## 4.1 Population

Twenty obese, type 2 diabetic Caucasian men (BMI>30 kg/m^2^), age 50-70 yr.

Twenty-two healthy obese Caucasian men (BMI>30 kg/m^2^), age 50-70 yr

Twenty-two healthy, lean Caucasian men (BMI18-25 kg/m^2^), age 50-70 yr

Subjects must meet all eligibility criteria for inclusion, including participation in a screening OGTT (not for diabetic subjects), to be able to exclude non-treated diabetics.

## 4.2 Screening visit

Before the screening, volunteers have to give their written informed consent. A general and medical questionnaire will be filled out, containing questions concerning our exclusion criteria. Fasting blood and urine samples will be collected and the following parameters will be determined for all volunteers:

- Fasting plasma glucose
- Fasting urinary glucose
- Fasting plasma insulin
- OGTT (Oral Glucose Tolerance Test)
- Length, weight and waist
- Blood pressure
- Hemoglobin levels

After the screening visit, the participants will receive a breakfast.

### 4.3 Inclusion criteria:

All subjects:

- Male sex
- Age 50-70 years
- Hemoglobin levels>8.4 mmol/L

For diabetic patients only:

- BMI > 30 kg/m2
- Must be on sulphonylurea- or metformin therapy for at least 6 months with a constant dose for at least two months, or on dietary treatment for at least 6 months
- Well-controlled diabetes: fasting plasma glucose concentration must be <10.0 mmol/l at the time of screening.

For obese controls only:

- BMI > 30 kg/m2
- normoglycemic according to WHO criteria (OGTT, fasting blood glucose< 7 mmol/L, after 2 hr <7.8mmol/L)
- Urine glucose concentrations <0,25 g/l
- systolic blood pressure <160 mmHg or diastolic blood pressure < 100 mmHg

For lean controls only:

- - BMI < 25 kg/m2
- normoglycemic according to WHO criteria (OGTT, fasting blood glucose< 7 mmol/L, after 2 hr <7.8 mmol/L)
- Urine glucose concentrations < 0,25 g/l
- systolic blood pressure <160 mmHg or diastolic blood pressure < 100 mmHg

## 4.4 Exclusion criteria

All subjects:

- Female gender
- Age below 50 or above 70 years
- Allergic to cow milk or dairy products
- Allergic to fish oil
- Vegetarian
- Tobacco smoker
- Current or recent (<4 weeks) use of fish oil supplements or more then four times fish/week; 24.35 g of EPA-DHA of fish per month (800 mg/day) as judged by the questionnaire.
- Received inoculations within 2 months of starting the study or planned to during the study
- Donated or intended to donate blood from 2 months before the study till two months after the study
- Unstable body weight (weight gain or loss > 3 kg in the past three months)
- Medical condition that can interfere with the study outcome (i.e. cardiovascular disease, gastrointestinal disease, renal dysfunction)
- Use of medications know to interfere with glucose homeostasis (i.e. corticosteroids)
- abuse of drugs and/or alcohol
- participation in another biomedical study within 1 month before the first screening visit

For obese, type 2 diabetic subjects only:

- severe diabetes which requires application of insulin
- diabetes-related complications

## 4.5 Sample size calculation

The main study outcome will be PBMC gene expression changes. In a previous study in which 21 healthy young males consumed high fat shakes (Bouwens et al., unpublished data), changes in expression of around 500 genes (using a false discovery rate correction) were detected in PBMC after consumption of the shakes. A changed expression of 500 genes is sufficient to perform pathway analysis.

Our study has a comparable design but we will include men aged between 50-70 yrs instead of healthy young adults. We expect that the magnitude of change in gene expression will be similar in this age group and will be higher in the risk profile groups.

Hence, we propose to include 20 subjects per group and to allow for a 10% dropout rate we will include 22 subjects per group.

# 5. INTERVENTION

##

## 5.1 Intervention meals

The high-fat shakes the participants will consume on each study day will contain 95 g fat each, and the total volume of each milkshake to be consumed will be 500 ml. The fatty acid composition is the only component that will differ between the milkshakes.

The basal constituent of all three milkshakes will be whipped cream and for every milkshake extra amounts of the specific fats of interest (i.e SFA, MUFA or PUFA) will be added. Water will be added to reach a total volume of 500 ml. For the composition of the milkshakes see table 2.

Table 2 : composition of the shakes

|  | | | **SFA shake** | **MUFA shake** | **PUFA shake** |
| --- | --- | --- | --- | --- | --- |
| Basal constituent (whipped cream) | | |  | | |
| Protein (g) | | | 3 | 3 | 3 |
| Carbohydrates (g) | | | 22 | 22 | 22 |
| Fat (g) | | | 40 | 40 | 40 |
| Extra added fat | | |  | | |
| Fat (g) | | | 55 | 55 | 55 |
|  | SFA (g) | | 45 | 10 | 10 |
|  | MUFA (g) | | 10 | 45 | 10 |
|  | PUFA (g) | | - | - | 35 |
|  |  | Of which EPA/DHA (g) | - | - | 22 |
| **Total** | | |  | | |
| Fat (g) | | | 95 | 95 | 95 |
| Energy (KJ) | | | 3938 | 3938 | 3938 |

## 5.2 Guidelines during the study period

All participants will be advised to keep the same dietary habits as before the study period. In order to standardize the participants‘ nutritional status before each testing day, the participants will be asked to:

- Consume a low fat evening meal (<10 g) the day before each study day (will be provided) and not to eat fish on the day before the study day
- Refrain from alcohol the day before each study day
- Avoid strenuous exercise the day before each study day
- Fast overnight before each study day (min of 10 hours).
- Transport to the university should be similar every study day

Details on how to live up to these guidelines will be given (and discussed) to each participant before the start of the study.

# 6. METHODS

## 6.1 Study outcomes

### 6.1.1 Measurements of primary study outcomes

PBMC gene expression

Gene expression changes will be assessed using whole genome Affymetrix microarrays for the samples at all time points.

### 6.1.2 Measurements of secondary study outcomes

PBMC immune response capacity

Cytokine profiles will be measured in plasma at all time points (T=0, 2 and 4 hrs) by quantitative immunoassays at Rules Based Medicine laboratories (Austin, Texas, USA). The cytokine profile of a panel of 120 proteins including adiponectin, CRP, and interleukins will be determined. For details on these 120 proteins, see: <http://www.rulesbasedmedicine.com/products-services/humanMAP-antigen.asp>

Plasma levels of additional potential biomarkers for metabolic syndrome (i.e. MBL2, FAM3D) will be measured in plasma by ELISA.

PBMC immune response will be determined by stimulation of the isolated cells ex vivo with lipopolysaccharide (LPS) for 4 hours. TNFα secreted in culture medium of the PBMC will be measured by ELISA. PBMC expression of genes involved in the acute immune response will be measured by qPCR.

Leukocyte activation markers will be determined at all time points (T=0, T=2, T=4 hrs) by using flow cytometry analysis (coulter Epics XL.MCL flow cytometry by Beckman Coulter).

Endothelial dysfunction

Macro vascular regional arterial stiffness will be assessed by Pulse Wave Analysis (PWA) (13). This is a non-invasive measurement which will be performed at every time point (T=0, 2 and 4 hrs).

A pressure sensor (applanation tonometer) is applied on the radial artery to record pressure pulse waveforms. The waveform is calibrated using systolic and diastolic pressure values from a conventional cuff measurement. From these peripheral waveforms we can derive a aortic pressure waveform and conduct a pulse wave analysis (PWA). A number of features can be extracted from a PWA, which cannot be made from the conventional measurement of brachial blood pressure. These features include the Augmentation Index (Aix) and the Subendocardial Viability Ratio (SEVR).

### 6.1.3 Measurements of tertiary study outcomes

Plasma lipids, glucose and insulin

Plasma lipid profile, glucose and insulin will be determined at all time points (T=0, T=2, T=4 hrs).

Monocyte and lymphocyte gene expression

T-cells and monocytes will be isolated via a negative selection procedure from PBMC using magnetic cell separation (MACS) tubes (Miltenyi Biotec). Gene expression changes in these cells will be assessed using whole genome Affymetrix microarrays for the samples at all time points.

Phenotype measurements

Body composition will be measured with the use of the BodPod.

MRI will be used to measure abdominal fat distribution, i.e. intra-abdominal fat and subcutaneous abdominal fat. In short, axial T1-weighted spin echo images will be acquired with a Philips Gyroscan NT Intera 1.0T scanner using the body coil with the patient in supine position. Slices will be centred at the interspace L4-L5, with the other slices situated above and below to cover the whole abdomen. A breath–hold technique will be applied to avoid breathing induced artefacts.

Measurements will be performed in collaboration with the Department of Radiology, Hospital Gelderse Vallei. All the measurements will be performed as much as possible by the same technician, and at the same time of the day. A experienced radiologist will evaluated all scans made

Fecal micro flora profiling will be done of feces collected at one time point in the study period.

## 6.2 Randomisation and blinding

Before the start of the study, participants of each ‘risk profile group’ will be randomly allocated to one of the six intervention groups (see table 1). The shakes will be labeled with three different codes. Participants will not know which code is allocated to which shake.

## 6.3 Study procedures

### 6.3.1 Information meeting

An information meeting will be organized 6 weeks before start of the study. In this meeting the investigators will explain the background, objectives and set up of the study. Also the possible risks and burdens will be explained and during this meeting there will be time for the potential participants to ask questions.

### 6.3.2 Screening

There will be a screening visit planned 4 weeks prior of the beginning of the study in order to recruit participants who fulfill all criteria. The following data will be collected:

- Written informed consent
- 3 ml of blood for fasting blood glucose
- Fasting urinary glucose concentrations
- Oral glucose tolerance test
- Length, weight and waist
- Blood pressure values
- A general and medical questionnaire will be filled out

### 6.3.3 Preparations prior to the Intervention

All participants will receive a diary 2 weeks prior to the study. In this diary, the participants can keep track of any signs of illness, any deviations from the protocol or any experienced side effects. In addition, further details (e.g. logistical) of the experiment will be elucidated.

### 6.3.4 Intervention

Figure 1 shows the study design and table 3 summarizes the assessments performed during the intervention.

Before the start of each study day, the Hb value of each participant will be determined by using a hemoglobin analyzer (HemoCue 201). Participants with a Hb value below 8.4 mmol/L will not be measured and are asked to make a new appointment. If Hb values are normal (e.g. above 8.4mmol/L) the measurements can be started. At T=-20 min, macro vascular regional arterial stiffness will be assessed and blood samples (62 ml) will be drawn by normal venopuncture. At T=0, the high-fat milkshake will be consumed within 15 min. Blood will be drawn 2 hours and 4 hours after intake of the shake and macro vascular regional stiffness will be assessed at the same time points.

The participants will spend the entire study duration (4.5 hours) in a room close to the blood collection room, where they will be able to read books, watch television, video or DVDs, or relax in comfortable chairs. At the end of the study day a lunch will be offered to the participants. At the end of the three study days the diaries will be collected*.*

SFA

SFA

SFA

MUFA

MUFA

MUFA

PUFA

PUFA

PUFA

SFA

SFA

SFA

MUFA

MUFA

MUFA

PUFA

PUFA

PUFA

SFA

SFA

SFA

MUFA

MUFA

MUFA

PUFA

PUFA

PUFA

Obese, diabetic subjects

Obese, healthy subjects

Lean, healthy subjects

Study day 1

Study day 2

Study day 3

Washout >1 wk

Washout >1 wk

Study day 1

Study day 2

Study day 3

T=0,2,4 hrs:

- Blood sampling

- Endothelial function measurement

T=0,2,4 hrs:

- Blood sampling

- Endothelial function measurement

T=0,2,4 hrs:

- Blood sampling

- Endothelial function measurement

Figure 1: Design of the study

Table 3: Protocol on each study day.

| **Intervention time, after 10h overnight fast** | **-20 min**  (7.00am) | **0 hr**  (7.20am) | **2 hr**  (9.20 am) | **4 hr**  (11.20 am) |
| --- | --- | --- | --- | --- |
| Consumption of high fat shake |  | x |  |  |
| Blood sampling (ml) | x |  | x | x |
| for plasma/serum isolation | 20 ml |  | 20 ml | 20 ml |
| for PBMC isolation | 42 ml |  | 42 ml | 32 ml |
| PBMC gene expression | x |  | x | x |
| PBMC stimulation with LPS | x |  | x |  |
| Cytokine profile | x |  | x | x |
| Plasma fatty acid profile | x |  | x | x |
| Plasma insulin and glucose | x |  | x | x |
| Endothelial function | x |  | x | x |
| Leukocyte activation markers | x |  | x | x |

## 6.4 Withdrawal of individual participants

Participants can leave the study at any time for any reason if they wish to do so without any consequences. The investigators can decide to withdraw a participant from the study for urgent medical reasons. After withdrawal of a participant, they will be replaced if this is still compatible with the study guidelines

## 6.5 Follow-up of participants after withdrawal

After possible withdrawal, no follow-up of participants will take place. In case of withdrawal due to medical complications, participants will be referred to a general physician.

## 6.6 Premature termination of the study

The procedures for premature termination of the study can be found in the adverse events protocol of the division Human Nutrition of Wageningen University.

# SAFETY REPORTING

## 7.1 Section 10 WMO event

When it appears that the disadvantages of participation may be significant greater than was foreseen in the research protocol, participants and the reviewing accredited METC will be informed according to section 10, subsection 1, of the WMO.

## 7.2 Adverse and serious adverse events

Adverse events (AE) are defined as any undesirable experience occurring to a participant during a clinical trial, whether or not considered related to the investigation. All AE reported by the participant or observed by the investigators will be recorded.

A serious adverse event (SAE) is any untoward medical occurrence or effect that at any dose results in death:

- Is life threatening (at the time of the event)
- Requires hospitalization or prolongation of hospitalization.
- Results in persistent or significant disabilities or incapacity
- Is a congenital anomaly
- Is a new event of the trial likely to affect the safety of the participants, such as an unexpected outcome of an adverse reaction, lack of efficacy of an IMP used for the treatment of a life threatening disease, major safety finding from a newly completed animal study, etc.

All SAE will be reported to the accredited METC that approved the protocol, according to the requirements of that METC.

## 7.3 Follow-up of adverse events

All AE will be followed until they have abated, or a stable situation has been reached. Depending on the event, follow-up may require additional test or medical procedures as indicated, and/or referral to the general physician or a medical specialist.

# STATISTICAL ANALYSIS

In this cross-over study, each subject will consume the three shakes and can be used as its own reference.

Analysis of PBMC whole genome expression data will be performed using a Bayesian linear regression model (implemented in the software package LIMMA; [www.bioconductor.org](http://www.bioconductor.org/)). Raw microarray data will be normalized using RMA quantile normalization. Statistically significant changed genes will be identified by student paired t-test followed by a false discovery rate correction (FDR) The latter is used to correct for multiple testing.

Analysis of secondary and tertiary outcome variables (endothelial function, glucose etc.) will be in SPSS version 15.0. Effects of treatment (SFA, MUFA, PUFA) within each risk group (lean healthy, obese, type 2 diabetic) will be tested by repeated measures ANOVA (mixed model) using pre-defined contrasts, i.e. 2 versus 0 hrs and 4 versus 2 hrs. Additionally, incremental areas under the curve will be calculated for all significant altered outcome variables using the trapezoïdal rule and compared between treatments using one-way ANOVA.

Differences between risk groups for each treatment will be tested by one-way ANOVA followed by LSD correction. Additionally, incremental areas under the curve will be calculated for all significant altered outcome variables and compared between risk groups using one-way ANOVA.

An empty ANOVA table providing more information about the analysis is included below.

| Variable | df |
| --- | --- |
| Risk profile (diabetic, obese, control) | 2 |
| Treatment (SFA, MUFA, PUFA) | 2 |
| Time (0,2,4) | 2 |
| Interaction | 8 |
| Error | 579 |

MRI scans will be analyzed using the HIPPO tool (14). Image files will be loaded into the HIPPO software and are then analyzed using the segment slice function. This software function initiates the automatic segmentation of the abdominal image into fat and non-fat components by designating each pixel as fat or non-fat. HIPPO is based on the fuzzy c-mean approach (i.e., fuzzy logic) and is able to make unsupervised classification of data into a number of clusters by identifying different tissues in an image without the use of an explicit threshold. HIPPO then automatically divides the pixels into light (fat) and dark (non-fat) and expresses them as a twin-peak histogram. The area under the peak representing fat will be expressed as a volume (centimeters cubed). The output result will be in the form of a computer spreadsheet.

# ETHICAL CONSIDERATIONS

## 9.1 Regulation statement

The study will be conducted according to the principles of the Declaration of Helsinki (Seoul, 2008) and in accordance with the Medical Research Involving Human Subjects Act (WMO).

## 9.2 Recruitment and consent

Subjects will be recruited from Wageningen and surrounding area. An invitation to participate will be sent by email to all eligible men in the database of participants of past scientific studies in the Division of Human Nutrition. If needed, additional recruitment of subjects will take place by advertisement in the University Weekly (Resource magazine) and local newspapers.

For the recruitment of obese (and diabetic) subjects an advertisement will be placed on the website of the Obesity Association ([www.obesitasvereniging.nl](http://www.obesitasvereniging.nl/)). Obese type 2 diabetic subjects will be informed about the study with the help of general practitioners.

Interested participants will be invited for an information meeting organized 6 weeks before the start of the study where the study, requirements for participation and risks will be explained by the Principal Investigator. An information booklet will be provided during this information meeting. Written informed consent will be obtained during a screening visit 4 weeks before the start of the study. In the informed consent we will ask the participants if they want to give permission for anonymous storage of their blood samples for possible additional analyses in the future. We will ask the METC for approval before performing additional analyses.

## 9.3 Privacy

Personal data will be stored in a closed locker and a password-protected file, to which only the investigators have access. Samples will be coded and destroyed within twelve years. Only the investigators have access to the code.

## 9.4 Benefits and risks assessment, group relatedness

### 9.4.1 Potential value of the research

The outcome of this study will provide us with valuable information about the postprandial response of high risk subjects to different fatty acids.

The collected data of PBMC gene expression, plasma cytokine profiles, endothelial function plasma lipid profile, glucose, insulin and activation markers of white blood cells could provide us knowledge about the molecular mechanisms behind development of postprandial dyslipidemia and endothelial dysfunction and the effect of different fatty acids on this process.

The immune stimulation experiment with LPS will provide us with more information about the immune response in obese and diabetic subjects and the effect of a high fat challenge on this.

### 9.4.2 Benefits for the participants

Participants that complete the study will receive €90. Participants are free to withdraw from further participation for any reason and at any time during the trial and will receive a proportional bonus for the effort they made (30 euro for each completed testing day). Participants can receive a summary of the results with averages of the group after the end of the study. Furthermore, participants will receive a meal the day before each study day and at the end of each study day.

### 9.4.3 Risks for the participants

There are minor risks for the participants during the intervention. The consumption of the high-fat shakes is not expected to be associated with any risk, although in rare cases it could lead to mild gastro-intestinal discomfort (belching, flatulence or loose stools). Participants also could experience a fatty taste in their mouth after intake of the high-fat shakes, and a fishy aftertaste in case of the PUFA enriched shake. Venapunctures can occasionally cause a local haematoma or bruise and some participants may report pain or discomfort.

The performed functional measurements of endothelial function are non-invasive and risks are therefore minimal.

MRI is a safe procedure, with no known health risk as long as no contraindication is met. These contraindications include persons with the following devices:

- Central nervous system aneurysm clips;
- Implanted neural stimulator;
- Implanted cardiac pacemaker or defibrillator;
- Cochlear implant;
- Ocular foreign body (e.g. metal shavings);
- Insulin pump;
- Metal shrapnel or bullet;
- Or metal containing corpora aliena in the eye of brains

MRI can result in a ‘coincidence’ finding. These findings should be reported and subjects will be informed about this.

Subjects that do not want to be informed about a ‘coincidence’ finding can not participate in the MRI measurement.

The time investment requested from the participants is shown in table 4.

Table 4: Time investment per participant

|  | Time at university/ hospital (OGTT, consumption of milkshakes, blood sampling etc) | Questionnaires | Weight/height /blood pressure | Blood collection during screening | Total |
| --- | --- | --- | --- | --- | --- |
| Information meeting | 1 hour |  |  |  | 1 hr |
| Screening | 2 hours | 15 min | 15 min | 10 min | 2.5 hrs |
| Week 1/2 | 4.5 hours |  |  |  | 4.5 hrs |
| Week 3/4 | 4.5 hours |  |  |  | 4.5 hrs |
| Week 5/6 | 4.5 hours |  |  |  | 4.5 hrs |
| MRI | 1 hour |  |  |  | 1 hr |
| Total time whole study | | | | | 18 hrs. |

## 9.5 Compensation for injury

Wageningen University has a liability insurance which is in accordance with article 7, subsection 6 of the WMO.

Wageningen University (also) has an insurance which is in accordance with the legal requirements in the Netherlands (Article 7 WMO and the Measure regarding Compulsory Insurance for Clinical Research in Humans of 23rd June 2003). This insurance provides cover for damage to research subjects through injury or death caused by the study.

1. € 450.000,-- (i.e. four hundred and fifty thousand Euro) for death or injury for each participant who participates in the Research;

2. € 3.500.000,-- (i.e. three million five hundred thousand Euro) for death or injury for all participants who participate in the Research;

3. € 5.000.000,-- (i.e. five million Euro) for the total damage incurred by the organization for all damage disclosed by scientific research for the Sponsor as ‘verrichter’ in the meaning of said Act in each year of insurance coverage.

The insurance applies to the damage that becomes apparent during the study or within 4 years after the end of the study.

Wageningen university has this insurance at:

Gerling-Konzern

Allgemeine Versicherungs-AG

Directie voor Nederland

Herengracht 520

1017 CC Amsterdam

# ADMINISTRATIVE ASPECTS AND PUBLICATION

## 10.1 Handling and storage of data and documents

Before the start of the study, participants will be assigned a random number that will not change during the study. This number is linked with the name, address, date of birth, and telephone number of the participant in a password-protected file. Only members of the project team can access this file. For all other purposes, the random number will be used for participant identification. The informed consents will be stored separately from all other information. After the end of the study the link between the identity of the participants and the assigned numbers will be destroyed. Only means and other statistical expressions based on individual data will be published. Human material, which is sampled during the study and stored anonymously, will be destroyed 12 years after the end of the study.

## 10.2 Amendments

Amendments are changes made to the research after a favorable opinion by the accredited METC has been given. All amendments will be notified to the METC that gave a favorable opinion.

## 10.3 End of study report

The investigator will notify the accredited METC of the end of the study within a period of 8 weeks. The end of the study is defined as the last day of the intervention. In case the study is ended prematurely, the investigator will notify the accredited METC, including the reasons for the premature termination.

## 10.4 Public disclosure and publication policy

Publication policy is in agreement with the CCMO publication statement. Nor the sponsors, nor the principal investigator has a right of veto regarding the way of publishing the results. Partners/ sponsors have the right to take cognizance of the results in order to patent some of the outcomes (if applicable).

# REFERENCES

1. van Oostrom AJ, Rabelink TJ, Verseyden C, et al. Activation of leukocytes by postprandial lipemia in healthy volunteers. Atherosclerosis 2004;177:175-82.

2. Jackson KG, Wolstencroft EJ, Bateman PA, Yaqoob P, Williams CM. Acute effects of meal fatty acids on postprandial NEFA, glucose and apo E response: implications for insulin sensitivity and lipoprotein regulation? Br J Nutr 2005;93:693-700.

3. Vogel RA, Corretti MC, Plotnick GD. Effect of a single high-fat meal on endothelial function in healthy subjects. Am J Cardiol 1997;79:350-4.

4. van Oostrom AJ, Sijmonsma TP, Verseyden C, et al. Postprandial recruitment of neutrophils may contribute to endothelial dysfunction. J Lipid Res 2003;44:576-83.

5. Valk PJ, Verhaak RG, Beijen MA, et al. Prognostically useful gene-expression profiles in acute myeloid leukemia. N Engl J Med 2004;350:1617-28.

6. Bomprezzi R, Ringner M, Kim S, et al. Gene expression profile in multiple sclerosis patients and healthy controls: identifying pathways relevant to disease. Hum Mol Genet 2003;12:2191-9.

7. Bouwens M, Afman LA, Muller M. Fasting induces changes in peripheral blood mononuclear cell gene expression profiles related to increases in fatty acid beta-oxidation: functional role of peroxisome proliferator activated receptor alpha in human peripheral blood mononuclear cells. Am J Clin Nutr 2007;86:1515-23.

8. Lopez-Miranda J, Williams C, Lairon D. Dietary, physiological, genetic and pathological influences on postprandial lipid metabolism. Br J Nutr 2007;98:458-73.

9. Blackburn P, Despres JP, Lamarche B, et al. Postprandial variations of plasma inflammatory markers in abdominally obese men. Obesity (Silver Spring) 2006;14:1747-54.

10. Nappo F, Esposito K, Cioffi M, et al. Postprandial endothelial activation in healthy subjects and in type 2 diabetic patients: role of fat and carbohydrate meals. J Am Coll Cardiol 2002;39:1145-50.

11. Stefan N, Kantartzis K, Machann J, et al. Identification and characterization of metabolically benign obesity in humans. Arch Intern Med 2008;168:1609-16.

12. Gibson GR, Roberfroid MB. Dietary modulation of the human colonic microbiota: introducing the concept of prebiotics. J Nutr 1995;125:1401-12.

13. Nichols WW ORM. McDonald’s blood flow in arteries. Theoretical, experimental and clinical principles. . 5th Edition. ed: Arnold, London, 2005.

14. Positano V, Gastaldelli A, Sironi AM, Santarelli MF, Lombardi M, Landini L. An accurate and robust method for unsupervised assessment of abdominal fat by MRI. J Magn Reson Imaging 2004;20:684-9.
